# Acquired von Willebrand syndrome in a patient with rectal adenocarcinoma: a case report and review of the literature

**DOI:** 10.1186/s13256-026-06234-1

**Published:** 2026-06-17

**Authors:** Stefano De Polo, Mariangela Costanza, Francesco Grandoni, Lorenzo Alberio, Gerasimos Tsilimidos

**Affiliations:** Service and Central Laboratory of Hematology, Lausanne University Hospital (CHUV) and UNIL, Rue du Bugnon 46, 1011 Lausanne, Switzerland

**Keywords:** Acquired von Willebrand syndrome, Rectal adenocarcinoma, Von Willebrand syndrome, Colorectal neoplasia

## Abstract

**Background:**

Acquired von Willebrand syndrome is a rare and often underdiagnosed entity, secondary to different pathologies, often of autoimmune or paraneoplastic etiology.

**Case presentation:**

Here, we report the first case of acquired von Willebrand syndrome secondary to rectal adenocarcinoma in a 62-year-old white patient with a negative history of a hemorrhagic diathesis. The patient received chemo-radiotherapy with tumor mass decrease and subsequent surgical removal. A pre-surgery workup identified isolated prolonged aPTT that led to coagulation factor analysis. Following prophylactic von Willebrand factor substitution, the surgery was performed without hemorrhagic complications. After treatment of the oncological disease, we observed spontaneous resolution of von Willebrand syndrome.

**Conclusions:**

It is important to recognize acquired von Willebrand syndrome because it can put the patient at a high risk of bleeding complications. Our case reports, for the first time, the presence of this syndrome secondary related to rectal adenocarcinoma. We also reviewed a few cases of acquired von Willebrand syndrome secondary to underlying solid tumors described in the literature.

## Background

Acquired von Willebrand syndrome (AVWS) is a bleeding disorder characterized by structural or functional defects of the von Willebrand factor (VWF) that is secondary to different underlying pathologies [1]. In particular, AVWS is frequently associated with cardiovascular disorders (congenital cardiac anomalies, left ventricular assist device, aortic stenosis, severe mitral regurgitation), lymphoproliferative disorders (monoclonal gammopathy of undetermined significance, multiple myeloma, chronic lymphocytic leukemia, Waldenström macroglobulinemia), myeloproliferative neoplasms (essential thrombocythemia, polycythemia vera, primary myelofibrosis), autoimmune disorders, hypothyroidism, certain solid malignancies (particularly Wilms tumor), and some medications such as valproate and ciprofloxacin [1–4].

The true prevalence of AVWS is unknown because many cases may be clinically silent or mild and remain undiagnosed. Underreporting is likely due to the lack of routine testing for individuals without bleeding.

Here, we report a case of AVWS associated with rectal adenocarcinoma and review recent literature findings. Signed informed consent was obtained from the patient included in this work.

## Case presentation

A 62-year-old white woman with no history of major surgery under treatment for hypertension, hypercholesterolemia, and hypothyroidism, with newly diagnosed rectal adenocarcinoma of the lower rectum of stage mrT3c mrN + EMVI + cM0, was scheduled for surgery after neoadjuvant treatment.

She received four cycles of neoadjuvant radio-chemotherapy according to the FOLFIRINOX (folinic acid, fluorouracil, irinotecan, and oxaliplatin) protocol and two cycles of FOLFOX (folinic acid, fluorouracil, and oxaliplatin) combined with local radiotherapy and capecitabine.

Following this treatment, a slight decrease in tumor volume and a reduction in the size of most mesorectal nodes were observed on MRI, and surgical resection was scheduled. The clinical examination revealed no abnormalities, except for a palpable rectal tumor 3 cm from the anal margin, with no infiltration of the external sphincter and a normal perianal condition with no signs of irritation.

Preoperative laboratory tests performed immediately before the operation, when the patient was already in the operating room, showed an isolated aPTT of 49 s (the aPTT reagent used was Pathromtin SL^®^; the Siemens Healthineers® machine was used; normal aPTT range 24–38 s). Unfortunately, no previous aPTT values are available. Further investigations in the hematology laboratory showed low VWF activity (16%) and a decreased VWF antigen level (33%) (VWF activity/antigen ratio of 0.5). The intrinsic pathway factors showed a factor VIII (FVIII) of 43%, normal factors IX and XI, and factor XII at 33%. The low VWF antigen level was considered the primary cause of the decreased factor VIII level; therefore, a mixing test to exclude an FVIII inhibitor was not considered necessary. The patient did not report any previous history of bleeding and did not have a family history of bleeding diathesis. In summary, these results raise the suspicion of AVWS.

The patient underwent Da Vinci robot-assisted abdominoperineal amputation with terminal colostomy. Given the previous results, we performed a rapid workup before surgery. She received 30 UI/kg (total 2000 units) of Wilate^®^ (containing VWF and FVIII in a 1:1 ratio) with concomitant tranexamic acid administration (a single administration of 1000 mg), with good biological recovery (VWF activity = 69% and FVIII = 75% at 30 min and VWF activity > 200% and FVIII = 250% at the end of surgery, activity at 24 h VWF activity 172% and FVIII = 140%) and no major or abnormal bleeding during or after surgery. After the initial surgery, the patient followed an adjuvant chemotherapy protocol with six cycles of 5-Fluorouracil, with complete remission of the tumor disease.

One month after the completion of adjuvant chemotherapy, coagulation tests were repeated. The investigations did not find any evidence of von Willebrand. More specifically, we found an aPTT of 43 s, factor XII of 51%, a normal von Willebrand activity of 106%, and an antigenic von Willebrand activity of 96%. After confirming the normalization of the values at a later time point (five months), we did not perform further analysis.

## Discussion

We initiated investigations due to a prolonged aPTT in the immediate preoperative period; no prior coagulation analysis had been performed.

As the patient had no history of bleeding (no prior hemostatic challenges characterized by bleeding) and given the urgency of the situation, we bypassed the mixing test. We proceeded directly to the analysis of individual coagulation factors, including VWF. We found a decreased factor XII level; however, such deficiencies are of no clinical significance and do not predispose to a bleeding diathesis. In our case, they can be explained by contact activation in the proinflammatory malignancy setting or by a congenital deficit. The decrease in factor VIII (43%) opened the differential diagnosis between von Willebrand disease (VWD), AVWS, and acquired hemophilia A (AHA). Given the pathological value of the activity-to-antigen VWF ratio (VWF:Ac 16%, VWF:Ag 0.5) and in the absence of a relevant history of hemorrhagic diathesis, our clinical hypothesis focused on AVWS. Hypothyroidism was well controlled without a recent change in substitution dosage; therefore, AVWS seemed unlikely to be attributable to thyroid disease, which was well controlled before the operation (TSH 0.98 mUI/l). The patient had normal hemoglobin, white blood cell, and creatinine values, and no history of symptoms suggestive of heart disease or lymphoproliferative or autoimmune disorders. Thus, our primary hypothesis was that AVWS is secondary to rectal adenocarcinomas. In the urgent preoperative setting, we administered Wilate^®^ (containing VWF and FVIII in a 1:1 ratio) with rapid recovery at 30 min. The high values at the end of the operation and at 24 h reflect the pharmacokinetics of Wilate^®^.

**Fig. 1:**
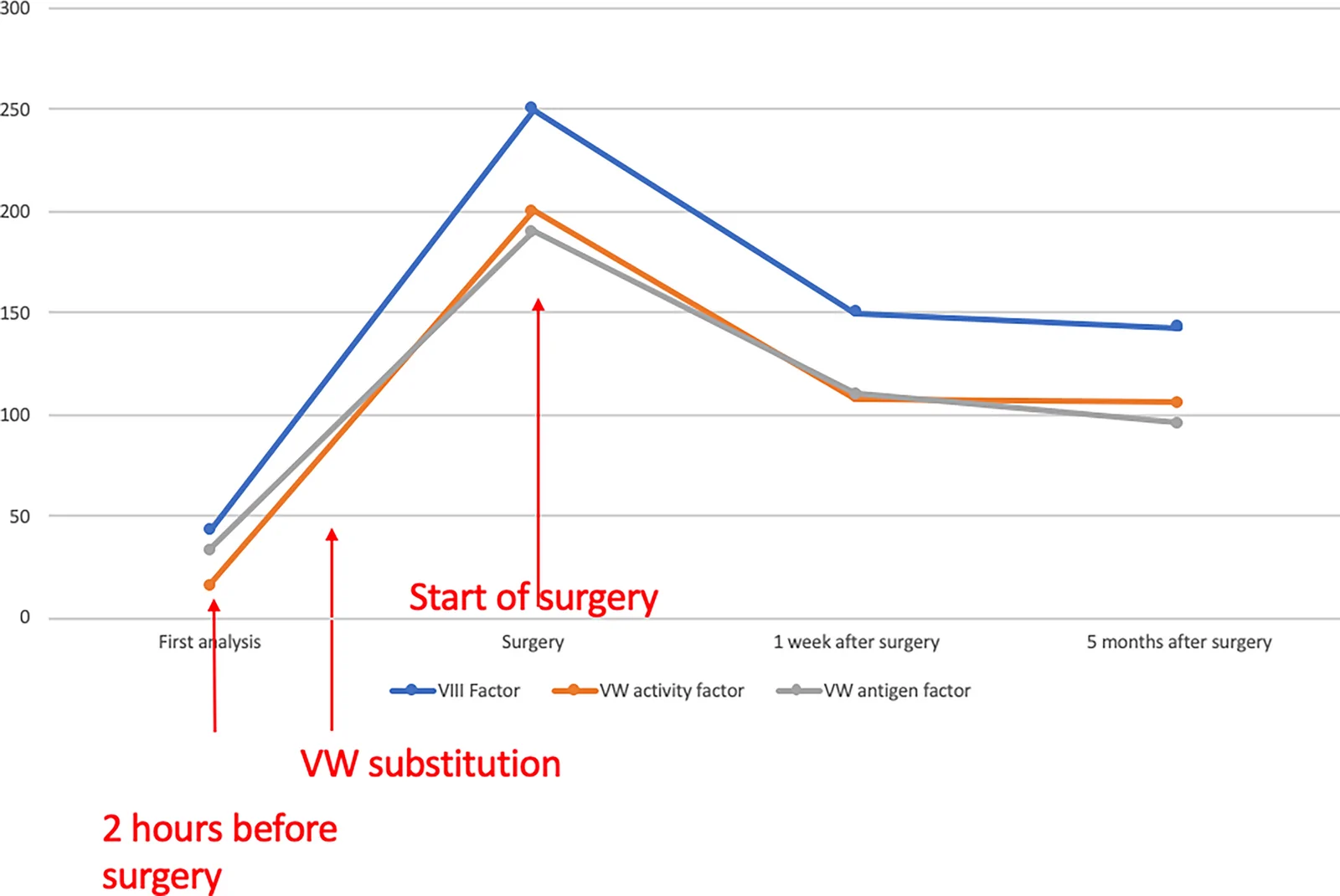
Graphic representing the variation of VIII factors, VW activity and VW antigen factors as a function of time: from the time of the first measurement to the follow-up after treatment

Given the rapid and persistent recovery of VWF values with remission of the adenocarcinoma, we did not perform any further analysis (Fig. 1), and we accepted this hypothesis as the definitive diagnosis. If the values had not been normalized, we would have completed the investigation by screening for MGUS, cardiac abnormalities, or autoimmune disorders, all of which can cause AVWS. The prolonged aPTT observed in our patient was partially attributable to Factor XII deficiency and to decreased FVIII activity.

AVWS is rarely associated with solid tumors. Data from the AVWS international register, published in 2000 by Federici [2], showed that this condition is secondary to a solid neoplasm in only 5% of cases. However, owing to the absence of major spontaneous bleeding in the absence of significant trauma, this condition is probably underdiagnosed in cases of invasive procedures or surgery.

Among solid tumors, AVWS has been reported to be associated with Wilms tumors [5], clear cell renal cell carcinoma [6], osteosarcoma [7], Ewing sarcoma [8], malignant peripheral neuroectodermal tumor [9], bladder cancer [10], epithelial myoepithelial carcinoma [11], and prostate and adrenal carcinoma [12, 13] (Table 1). To our knowledge, this is the first reported case of AVWS secondary to gastrointestinal adenocarcinoma.

**Table 1:** Published cases of AVWS in the context of a solid tumor

Type of tumor	N° of cases	References
Wilms tumor	8	Fosbury et al. [5]
Clear cell renal cell carcinoma	1	Odom et al. [6]
Osteosarcoma	1	Agrawal et al. [7]
Ewing sarcoma	1	Elli et al. [8]
Malignant peripheral neuroectodermal tumor	1	Nowak-Göttl et al. [9]
Prostate carcinoma	1	Claus et al. [12]
Adrenal carcinoma	1	Facon et al. [13]
Bladder cancer	1	Dumas et al. [10]

There are several known mechanisms responsible for the decrease in VWF associated with immune and non-immune mechanisms; in some conditions, several mechanisms may coexist [1]. Immune-mediated reduction of VWF by specific and non-specific autoantibodies may occur in lymphoproliferative disorders, myeloproliferative neoplasms, and autoimmune diseases [14]. The non-immune-mediated mechanisms include the following: (a) increased degradation of VWF multimers by shear stress-induced proteolysis [15, 16] or by other proteases, (b) decreased synthesis [17], and (c) adsorption onto malignant cells or proteins [18].

The pathophysiological mechanisms of solid tumors have been elucidated only for Wilms tumors and appear to be linked to hyaluronic acid secretion by nephroblastoma cells. In vitro studies have shown that elevated levels of hyaluronic acid interfere with the formation of the von Willebrand factor/factor VIII complex [19, 20].

The mechanisms underlying the decrease in VWF in our case and in other solid tumor types remain unclear due to a lack of more detailed studies. No studies have used VWF-propeptide levels to clarify the underlying pathogenetic mechanism of AVWS. In our case, during oncologic follow-up, local disease recurrence was diagnosed in the absence of VWF abnormalities. This observation led us to hypothesize that VWF adsorption may play a role, since, under this mechanism, a lower tumor burden may not be sufficient to decrease VWF levels significantly. However, this mechanism was not evaluated in the histological analysis of our patient (Fig. 1).

The treatment of the underlying cause is the only potentially curative option for AVWS. However, this is not always possible. In our case, following rectal adenocarcinoma removal, we observed normalization of VWF. When complete remission cannot be achieved, prevention of high-risk situations and management of acute bleeding are important [1].

## Conclusions

In summary, AVWS should be considered in patients with solid tumors who present with unexplained, clinically relevant bleeding or abnormal global coagulation assays. Although rare, this disease can put patients at a high risk of bleeding complications during surgery. Further studies are needed to elucidate the mechanisms underlying the development of AVWS and to guide treatment in acute and follow-up settings.

## Data Availability

Data sharing is not applicable to this article as no data were created or analyzed in this study.

## Abbreviations

AVWS: Acquired von Willebrand syndrome
VWD: Von Willebrand disease
VWF: Von Willebrand factor
VW: Von Willebrand
FVIII: Factor VIII
AHA: Acquired hemophilia A

## References

[1] Franchini M, Mannucci PM. Acquired von Willebrand syndrome: focused for hematologists. Haematologica. 2020;105:2032–7. 32554559 10.3324/haematol.2020.255117 PMC7395262

[2] Federici AB, *et al*. Acquired von Willebrand syndrome: data from an international registry. Thromb Haemost. 2000;84:345–9. 10959711

[3] Kreuz W, *et al*. Valproate therapy induces von Willebrand disease type I. Epilepsia. 1992;33:178–84. 1733754 10.1111/j.1528-1157.1992.tb02303.x

[4] Castaman G, Lattuada A, Mannucci PM, Rodeghiero F. Characterization of two cases of acquired transitory von willebrand syndrome with ciprofloxacin: evidence for heightened proteolysis of von willebrand factor. Am J Hematol. 1995;49:83–6. 7741144 10.1002/ajh.2830490114

[5] Fosbury E, Szychot E, Slater O, Mathias M, Sibson K. An 11‐year experience of acquired von Willebrand syndrome in children diagnosed with Wilms tumour in a tertiary referral centre. Pediatr Blood Cancer. 2017;64: e26246. 10.1002/pbc.26246 27616321

[6] Odom B, Khourdaji I, Golas V, Zekman R, Rosenberg B. Acquired von Willebrand disease secondary to clear cell renal cell carcinoma. J Endourol Case Rep. 2018;4:114–6. 30065959 10.1089/cren.2018.0032 PMC6056257

[7] Agrawal AK, Golden C, Matsunaga A. Acquired von Willebrand disease in an osteosarcoma patient. J Pediatr Hematol Oncol. 2011;33:622–3. 22042279 10.1097/MPH.0b013e31821d80be

[8] Elli M, *et al*. Acquired von willebrand syndrome in a patient with Ewing sarcoma. Pediatr Hematol Oncol. 2006;23:111–4. 16651239 10.1080/08880010500457749

[9] Nowak-Göttl U, *et al*. Acquired von willebrand disease in malignant peripheral neuroectodermal tumor (PNET). Med Pediatr Oncol. 1995;25:117–8. 7603396 10.1002/mpo.2950250213

[10] Dumas G, Rousseau B, Rodrigues MJ, Tlemsani C, Goldwasser F. Acquired Type II von Willebrand Syndrome in Locally Advanced Bladder Cancer Successfully Treated With Intravenous Immunoglobulin and Chemotherapy. Clin Genitourin Cancer. 2016;14:e95–7. 26362071 10.1016/j.clgc.2015.07.013

[11] Chilvers, G. S. & Porter, G. Acquired von Willebrand’s disease associated with epithelial myoepithelial carcinoma of the parotid salivary gland. *Case Rep.* **2014**, bcr2014205248–bcr2014205248 (2014). 10.1136/bcr-2014-205248 PMC4127676 25096657

[12] Claus P-E, Van Haute I, Verhoye E, Deeren D, Moreau E. Diagnostic challenges in acquired von Willebrand syndrome: a complex case of prostate carcinoma associated-acquired von Willebrand syndrome. Semin Thromb Hemost. 2016;43:101–4. 27806384 10.1055/s-0036-1592167

[13] Facon T, *et al*. Acquired type II von Willebrand’s disease associated with adrenal cortical carcinoma. Br J Haematol. 1992;80:488–94. 1581233 10.1111/j.1365-2141.1992.tb04562.x

[14] Mannucci PM, *et al*. Studies of the pathophysiology of acquired von Willebrand’s disease in seven patients with lymphoproliferative disorders or benign monoclonal gammopathies. Blood. 1984;64:614–21. 6432075

[15] Heilmann C, *et al*. Acquired von Willebrand syndrome in patients with extracorporeal life support (ECLS). Intensive Care Med. 2012;38:62–8. 21965100 10.1007/s00134-011-2370-6

[16] Tauber H, *et al*. Extracorporeal membrane oxygenation induces short-term loss of high-molecular-weight von Willebrand factor multimers. Anesth Analg. 2015;120:730–6. 25565317 10.1213/ANE.0000000000000554

[17] Dalton RG, *et al*. Hypothyroidism as a cause of acquired von Willebrand’s disease. Lancet Lond Engl. 1987;1:1007–9. 10.1016/s0140-6736(87)92272-0 2883346

[18] Richard C, *et al*. Acquired von Willebrand disease in multiple myeloma secondary to absorption of von Willebrand factor by plasma cells. Am J Hematol. 1990;35:114–7. 2205095 10.1002/ajh.2830350210

[19] Michiels JJ, Schroyens W, Bememan Z, van der Planken M. Atypical variant of acquired von willebrand syndrome in wilms tumor: is hyaluronic acid secreted by nephroblastoma cells the cause? Clin Appl Thromb. 2001;7:102–5. 10.1177/107602960100700204 11292185

[20] Bracey AW, *et al*. Platelet dysfunction associated with wilms tumor and hyaluronic acid. Am J Hematol. 1987;24:247–57. 3030095 10.1002/ajh.2830240304

